# Circular RNA VMA21 ameliorates lung injury in septic rat via targeting microRNA-497-5p/CD2-associated protein axis

**DOI:** 10.1080/21655979.2022.2031406

**Published:** 2022-02-16

**Authors:** JinFang Ke, MengFei Chen, ShiLan Ma, Liang Zhang, Ling Zhang

**Affiliations:** Department of Emergency, People’s Hospital of Ningxia Hui Autonomous Region, YinChuan City, NingXia Hui Autonomous Region, China

**Keywords:** Circular RNA RNAVMA21, MicroRNA-497-5p, CD2-associated protein, sepsis, lung injury

## Abstract

Sepsis was characterized via an acute inflammatory response to infection, often accompanying by multiple organ failure, particularly lung damage. Circular RNA (circRNA) played an important role in the pathology of a variety of diseases. However, the role of circRNA in sepsis-induced lung injury (LI) remained unknown. This study was to explore the expression and role of circVMA21 in sepsis LI and the possible molecular mechanism. The results manifested circVMA21 and CD2-associated protein (CD2AP) were down-regulated in lung tissue and lipopolysaccharide (LPS)-treated BEAS-2B, while microRNA (miR)-497-5p was up-regulated. A large number of deaths in rats after surgery of 72 h were caused via cecal ligation-perforation surgery, W/D value and Bax positive cells were increased, LI was caused, cell apoptosis, tumor necrosis factor-α, Interleukin (IL)-1β and IL-6 expression were promoted and Bcl-2 positive cells were decreased. Overexpression of circVMA21 ameliorated these phenomena. In addition, LPS-induced apoptosis and inflammation of BEAS-2B cells was improved via overexpression of circVMA21, while overexpression of miR-497-5P was opposite. Apoptosis, inflammation, and oxidative damage of BEAS-2B cells were aggravated via knockdown of circVMA21, but it was reversed by knockdown of miR-497-5p or overexpression of CD2AP. Mechanistically, CircVMA21 mediated CD2AP expression through competitive adsorption of miR-497-5p. In conclusion, this work showed circVMA21 improved LI in sepsis rats by targeting miR-497-5p/CD2AP axis, suggesting that circVMA21 may be a novel therapeutic target for sepsis-induced LI.

## Graphical abstract

**Figure:**
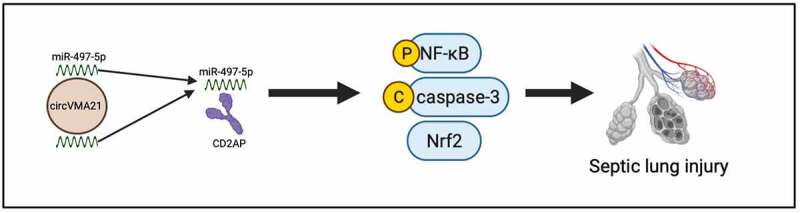

## 1 Introduction

Sepsis, a systemic inflammation syndrome, is available to trigger multiple organ dysfunction and is the crucial reason of acute lung injury (ALI) [1]. Sepsis takes up approximately 20% of all deaths in the world in line with the incomplete statistics [2]. Huge progression is in early resuscitation, organ protection treatment strategies and antibiotic treatment over the past decades, and the morbidity and mortality of sepsis are declined, but it remains a gigantic challenge for medical professionals [3].

As is well known, the lung is the major organ for gas exchange in mammals and a vital immune organ [4]. The lung is the major target organ for multiple pathogens, allergens and toxic substances that lead to pneumonia, acute respiratory distress syndrome and ALI or inflammation owing to direct contact with the external environment [5]. As reported, the lung is the susceptible organs of sepsis, while ALI is the crucial reason of death in sepsis patients [6]. The lung endothelial barrier is massively destroyed during sepsis-stimulated lung injury (LI), leading to pulmonary edema, refractory hypoxemia and an influx of pro-inflammatory white blood cells [7]. Foregoing studies have been clarified that a vital pathological feature of sepsis is the imbalance of host immune response. Aberrant physiological conditions are regarded as the conversion of effective linear splicing of protein-coding RNA to atypical splicing, which is primarily manifested via aberrant circular RNA (circRNA) [8].

CircRNA, a specific type of non-coding RNA, exerts a crucial action in the pathological process of multiple illnesses [9]. Numerous evidences elucidate that circRNA modulates gene during the progression of sepsis-stimulated LI [10]. For instance, circular RNA (circRNA) C3P1 declines the generation of pro-inflammatory cytokines and apoptosis in ALI stimulated via sepsis via modulating miR-21 [11]. CircVMA21, deriving from the vacuolar adenosine triphosphatase (ATPase) assembly factor, is a newly discovered circRNA [12]. Studies have been clarified that circVMA21 was silenced in sepsis-stimulated acute kidney injury (AKI), and elevation of circVMA21 was able to effectively ameliorate sepsis-stimulated AKI. Nevertheless, its action in sepsis-stimulated LI was unknown.

In this study, the function of circVMA21 in sepsis-stimulated LI was figured out in *vivo* and in *vitro*. Additionally, circVMA21ʹs influence on inflammatory cytokines, lung tissue injury and apoptosis and the underlying mechanism of circVMA21 were explored. These findings were supposed to offer novel treatment strategies for LI from sepsis.

## 2 Materials and methods

### 2.1 Construction of cecal ligation-perforation (CLP) model

Eighty males Sprague-Dawley rats (200–250 g, 7–8 weeks old) were performed (all the Laboratory Animal Center, Huazhong University of Science and Technology, Wuhan, China). Feeding of the rats was in an environment with giving adequate food and water. After one week of adaptive feeding, division of the rats was randomly into four groups (n = 20): The Sham, the CLP, the Ad-oe-NC, and the Ad-oe-circVMA21. Ligation and puncture (CLP) were conducted as previously for stimulation of sepsis [13]. The rats were fasted prior to surgery. Anesthesia of the rats was via intraperitoneal injection of sodium pentobarbital (40 mg/kg). In short, the abdominal skin was cut about 2 cm, and the cecum was gently pulled out with sterile forceps. Ligation of the root of the cecum was with 3–0 silk thread, and a hole was punched in the head and tail of the cecum. The distance between the two perforations was approximately 1 cm. Subsequently, Bits of feces was lightly extruded, reposition of the cecum in the abdominal cavity was conducted, and suture of the muscle, skin, and peritoneum was exerted. Rescue of the rats was immediately via subcutaneous injection of saline after the operation. CLP was not performed in the sham operation (Sham), and the rest of the steps were the same as the experiment. Injection of 20 μL Ad-oe-circVMA21 adenovirus solution (10^7^ particles/μL, RiboBio Co., Ltd., Guangzhou, China) was via tail vein one week prior to CLP surgery to elevate circVMA21. Injection of another group of rats was with the same dose of Ad-oe-NC adenovirus solution as a control. Behind CLP, test of 10 rats in each group was performed for 72 h survival. Euthanasia of the remaining rats in each group was conducted, and lung tissues were collected. Fixation of half of the lung tissues was in 4% paraformaldehyde for histopathological observation, and storing of the other half of the lung tissues was for subsequent protein and RNA extraction.

### 2.2 Dry-to-wet ratio

After taking the middle lobe of the right lung of the experimental rat, record of the wet weight (W) was after sucking up the surface water. Then heating of the middle lobe of the right lung was in an oven to constant weight, weight was performed repetitively and record was conducted as the dry weight (D). Pulmonary edema index = W/D.

### 2.3 Hematoxylin-eosin, TdT-mediated dUTP-biotin nick end-labeling (TUNEL) staining and histological damage scores

Embeddedness of the fixed lung tissue was in paraffin section (4 μm). Dehydration of the slices was with gradient alcohol, removal was with xylene, and seal was with resin. Staining of the sections was with hematoxylin and eosin solutions (Beyotime, Shanghai, China). Ultimately, monitoring of the pathological variation of the lung tissue was with a microscope (Olympus BX 53 microscope, Tokyo, Japan). Staining of the tissue sections was with TUNEL reagent (Roche, Basel, Switzerland) to test cell apoptosis on the grounds of the manufacturer’s instructions, and observation was performed adopting a fluorescence microscope (magnification 400 ×). LI scores were performed as previously described [14]. LI (including bleeding, inflammation, and edema) was graded on the following scale: 0 = normal, 1 = mild, 2 = moderate, 3 = severe and 4 = severe. The grading was performed by pathologists who were unaware of the experiment.

### 2.4 Immunohistochemistry

Immunohistochemical staining was performed as previously described [15]. Dewaxing of the sections was with xylene, hydration was with gradient alcohol, and block was with 3% H_2_O_2_ for inactivation of endogenous peroxidase. Behind blocking with 5% bovine serum albumin, treatment of the sections was with primary antibodies Bax (ab32503, Abcam) and Bcl-2 (ab32124, Abcam). Dropwise addition of the secondary antibody Goat Anti-Rabbit IgG (1:500, ab150077, Abcam, USA) was performed; Incubation of the sections was conducted. Dehydration of the sections was performed, removal was conducted, and fixation was on slide with a neutral resin. Ultimately, a microscope (Olympus BX 53 microscope, Tokyo, Japan) was performed.

### 2.5 Enzyme-linked immunosorbent assay (ELISA)

Extraction of total protein from lung tissues and cells was with radio-Immunoprecipitation assay (RIPA) buffer covering protease inhibitors, and test of pro-inflammatory factors tumor necrosis factor-α (TNF-α), Interleukin (IL)-1β, and IL-6 was on the basis of the manufacturer’ instructions. Observation of optical density (OD) at 450 nm was with a Power Wave microplate reader (Bio-TEK, USA). ELISA kits were performed (all MyBioSource Inc., San Diego, California, USA).

### 2.6 Cell culture

Maintaining of the human lung epithelial cell line BEAS-2B (all the American Type Culture Collection, ATCC; Manassas, VA, USA) was in Dulbecco’s Modified Eagle Medium replenished with penicillin (10 U/mL), 10% fetal bovine serum, and streptomycin (10 μg/mL). Placing of all cells was in a sterile incubator.

### 2.7 Cell transfection and lipopolysaccharide (LPS) exposure

Design and provision of the small interfering RNA targeting circVMA21 and CD2-associated protein (CD2AP) (si-circVMA21/CD2AP) and the elevation plasmid vector (pcDNA 3.1-circVMA21/CD2AP), miR-497-5p mimic/inhibitor with the corresponding negative control were performed (all RiboBio Co., Ltd., Guangzhou, China). Transfection of the above reagents was into BEAS-2B cells adopting Lipofectamine 2000 (Invitrogen) in the light of the manufacturer’s requirements. Culture of BEAS-2B cells was in medium with diverse doses of LPS (0.1, 1, 10, or 100 ng/mL) to simulate sepsis injury in *vitro*. Treatment of BEAS-2B cells was with 100 ng/mL LPS after transfection to explore the influence of gene gain or loss on sepsis.

### 2.8 3-(4, 5-dimethylthiazol-2-yl)-2, 5-diphenyltetrazolium bromide (MTT) assay for assessment of cell viability

Assessment of cell viability was in line with the foregoing methods [16]. In short, seeding of the cells was in 96-well plates, which was incubated with MTT solution (5 mg/mL, Sigma). Shift of 200 μL dimethyl sulfoxide solution was into each well for dissolution of the crystal formazan. Measurement of the absorbance was with a Power Wave microplate reader (Bio-TEK, USA) for assessment of cell viability.

### 2.9 Examination of apoptosis via flow cytometry

Cell apoptosis was assessed as previously described [17]. The cells were collected, and fixation was with 70% ethanol. Subsequently, double staining was adopting 10 μL Annexin V-fluoresceinisothiocyanat (FITC) and 5 μL propidium iodide (PI) to assess cell apoptosis. Carrying out of all solutions was on the grounds of Annexin-V-FITC Apoptosis Detection Kit (Beyotime, Shanghai, China). Analysis of the cells was through flow cytometry (BD Biosciences, San Jose, CA, USA) to quantify apoptosis.

### 2.10 Reverse transcription quantitative polymerase chain reaction (RT-qPCR)

Extraction of total RNA was adopting TRIzol LS reagent (Invitrogen), and reverse transcription was into cDNA adopting ReverTra Ace qPCR RT kit (Toyobo, Osaka, Japan) on the basis of the manufacturer’s instructions. Separation of the target miRNA was adopting miRVana™ miRNA isolation kit (Invitrogen). Performance of the reverse transcription of miRNA was via adopting All-in-One™ miRNA RT-qPCR detection kit (GeneCopoeia Inc., Rockville, USA). Amplification of the target gene was performed exerting the 7300 real-time PCR system (Thermo Fisher Scientific). Manifestation of miRNA and mRNA was with U6 and glyceraldehyde-3-phosphate dehydrogenase (GAPDH) as standard, separately. Calculation of the gene was adopting 2^−ΔΔCt^ equation. The primer sequences were presented in Table 1.

**Table 1.** PCR primer sequence

	Primer sequence (5′ – 3′)
CircVMA21	F: 5’- GCTGGCCCTCTTTGTGTATG-3’
	R: 5’- AATCCTGTTTGCCTTCACGC-3’
MiR-497-5p	F: 5’-CCTTCAGCAGCACACTGTGG-3’
	R: 5’- CAGTGCAGGGTCCGAGGTAT-3’
CD2AP	F: 5’- CTGTCAGCTGCAGAGAAGAAA −3’
	R: 5’- TTGGGTTGGAGAATGTCCAC-3’
GAPDH	F: 5’- CTGCCAACGTGTCAGTGGTG-3’ R: 5’- TCAGTGTAGCCCAGGATGCC-3’
U6	F: 5’- CGAATTTGCGTGTCATCCTT-3’
	R: 5’- CGAATTTGCGTGTCATCCTT-3’

F: Forward; R: Reverse

### 2.11 Western blot

Extraction of the total protein was with frozen RIPA buffer, and quantification was with the BCA protein detection kit (Pierce, Rockford, IL, USA). The same amount of protein (30 μg) was subjected to 12% sulfate polyacrylamide gel electrophoresis, and shift was to the nitrocellulose membrane. Adoption of 5% skimmed milk was to guard against nonspecific binding. Incubation of the membrane with primary antibodies cleaved caspase-3 (9661, Cell Signaling Technology), caspase-3 (9665, Cell Signaling Technology), p-NF-κB (3033, Cell Signaling Technology), NF-κB (4764, Cell Signaling Technology), and Nrf2 (ab62352, Abcam) was performed. Subsequently, incubation of the membrane was with a secondary antibody conjugating with horseradish peroxidase, and treatment of the membrane was with ECL reagent (Beyotime) to visualize the immune response band. Quantification of these bands was adopting Gel Doc™ XR imaging system (Bio-Rad Laboratories, Hercules, CA, USA) and ImageJ software. Adoption of GAPDH was as a loading control gene.

### 2.12 The luciferase activity assay

Reporter plasmids (luc-circVMA21-wt and luc-circVMA21-mut) covering wild-type (WT) or mutant (MUT) circVMA21 sequences, and reporter plasmids (luc-CD2AP-wt and luc-CD2AP-mut) involving WT or MUT CD2AP 3ʹuntranslated region sequence were synthesized (all Shanghai Gene Pharmaceutical Co., Ltd., Shanghai, China). When BEAS-2B cells were converged in 70%, co-transfection of the reporter plasmid and miR-497-5p mimic or mimic NC was into BEAS-2B cells in line with the manufacturer’s method adopting Lipofectamine 2000 (Invitrogen). Measurement of the luciferase activity was adopting the dual-luciferase reporter system (Promega), and comparison was with the Renilla luciferase activity in line with the manufacturer’s agreement.

## 2.13 RNA immunoprecipitation (RIP) experiment

RIP experiments was performed adopting Magna RNA Binding Protein Immunoprecipitation Kit (Millipore, Billerica, MA, USA) [18]. In short, incubation of the cell lysate was with RIP buffer covering magnetic beads conjugating with human anti-Ago2 antibody, or adoption of normal mouse IgG was as a reverse control. Incubation of the sample was with proteinase K, and separation of the immunoprecipitated RNA was conducted. Measurement of the RNA concentration was with a spectrophotometer (Thermo Scientific, Waltham, MA, USA), and assessment of the RNA quality was with a bioanalyzer (Agilent, Santa Clara, CA, USA). Additionally, extraction of purified RNA was performed; Analysis was via quantitative real-time PCR to prove the binding target.

### 2.14 Data analysis

Representation of all data was as mean ± standard deviation (SD). Adoption of statistical analysis and graphing was with GraphPad Prism v9.0 (GraphPad Software, Inc.). Comparison of the two was adopting the unpaired Student’s t test. The comparison of the multiple was adopting one-way analysis of variance (ANOVA) with Tukey’s multiple-comparison test. N = 3. *P* < 0.05 was accepted as indicative of significant differences.

## 3 Results

### 3.1 Elevation of circVMA21 ameliorates CLP-stimulated LI in rats

**Figure 1.**
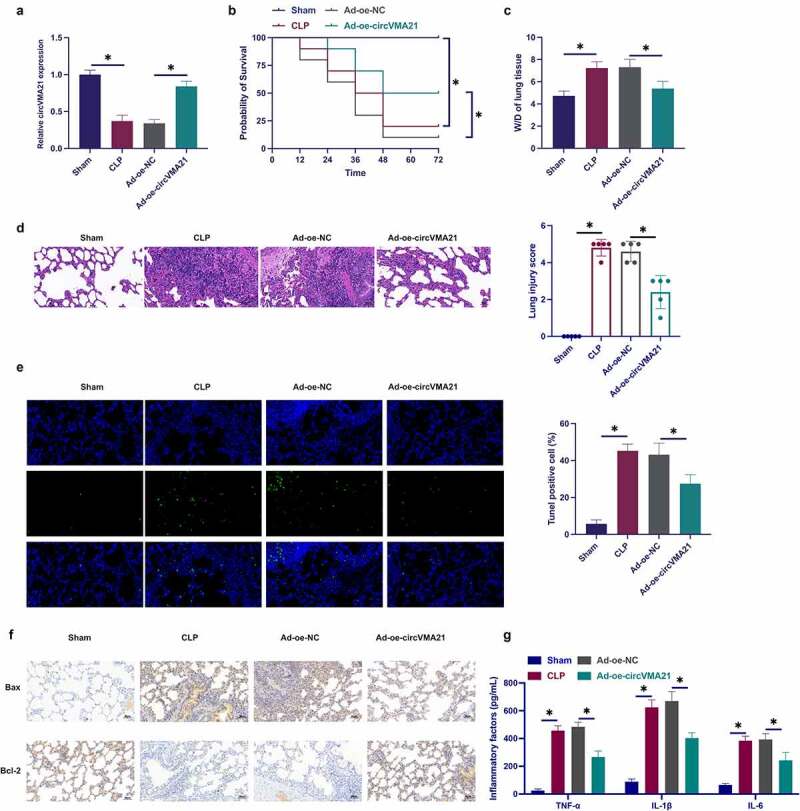
Elevation of circVMA21 ameliorates CLP-stimulated LI in rats. (a): Test of circVMA21 in rat lung tissue was via RT-qPCR; (b): Monitoring of 72 h survival rate of rats after CLP surgery was performed; (c): W/D of rat lung tissue after CLP surgery; (d): Representative images of HE staining in rat lung tissue; (e): Examination of apoptotic cells in rat lung tissue was via TUNEL staining; (f): Test of rat lung tissue Bax and Bcl-2 positive cells in rat lung tissue was via Immunohistochemistry; (g): Examination of the inflammatory factors in rat lung tissue was via ELISA; Representation of the data was as mean ± SD, n = 10; * *P* < 0.05.

Foregoing studies have been clarified that circVMA21 is aberrant in septic kidney tissue [19]. Nevertheless, the action of circVMA21 in septic LI was unknown. Construction of a rat model of sepsis was performed via CLP surgery, and circVMA21 in CLP rats was elevated via adenovirus injection. CLP surgery declined circVMA21 in lung tissue, while circVMA21 was distinctly recovered via the injection of shRNA adenovirus vector targeting circVMA21 (Figure 1(a)). The rat survival was monitored after CLP procedure. The mortality rate of CLP rats was 80% after operation of 72 h, while the overexpression of circVMA21 effectively reduced the mortality rate of CLP rats (Figure 1(b)). Analysis of pulmonary edema was via W/D test. CLP surgery augmented the W/D of rats, while elevation of circVMA21 weakened this effect, as proved in Figure 1c. CLP surgery led to severe alveolar damage, thickening of alveolar walls and diaphragms with vascular congestion in rats, while elevation of circVMA21 weakened this pathological tissue variation (Figure 1(d)). CLP surgery elevated the apoptosis rate of lung tissue cells, while augmentation of circVMA21 declined the apoptosis rate of lung tissue cells (Figure 1e). CLP elevated the number of Bax-positive cells in rat lung tissue, while the number of Bcl-2-positive cells were declined, and augmentation of circVMA21 weakened the variation of these two proteins (figure 1f). Test of the variation of lung tissue pro-inflammatory cytokines was performed. CLP distinctively augmented inflammation in lung tissue, but elevated circVMA21 declined it, as proved in Figure 1g. These data elucidated that augmentation of circVMA21 effectively ameliorated CLP-stimulated ALI.

### 3.2 Elevation of circVMA21 ameliorates LPS-stimulated apoptosis and inflammation of BEAS-2B cell

**Figure 2.**
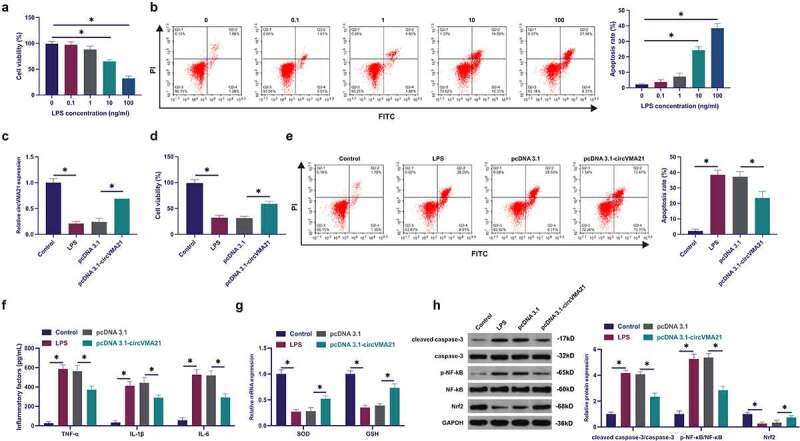
Augmentation of circVMA21 ameliorates LPS-stimulated apoptosis and inflammation of BEAS-2B cell. (a): Test of the influence of diverse concentrations of LPS treatment on the viability of BEAS-2B cells via MTT; (b): Examination of the influence of different concentrations of LPS treatment on the apoptosis rate of BEAS-2B cells was via flow cytometry; (c): Test of pcDNA 3.1- circVMA21 transfection efficiency via RT-qPCR; (d): Detection of the influence of elevation of circVMA21 on the viability of BEAS-2B cells was via MTT; (e): Examination of the impact of circVMA21 augmentation on the apoptosis rate of BEAS-2B cells via flow cytometry; (f): Test of the impact of circVMA21 augmentation of BEAS-2B cells inflammatory cytokines was via ELISA; (g): Examination of the influence of circVMA21 elevation on the antioxidant enzymes in BEAS-2B cells was via RT-qPCR; (h): Examination of the impact of circVMA21 augmentation on cleaved caspase-3, p-NF-κB and Nrf2 in BEAS-2B cells via Western blot; Representation of the data was as mean ± SD, N = 3; * *P*< 0.05.

Exploration of circVMA21ʹs action and mechanism in septic LI was conducted via adopting in *vitro* experiments. Construction of a model of BEAS-2B cell injury was via LPS treatment with different concentrations. LPS declined the cell viability of BEAS-2B cells, and accelerated apoptosis rate dose dependently, as proved in Figure 2(a,b). Subsequently, circVMA21 in BEAS-2B cells was elevated via transfection of pcDNA 3.1 targeting circVMA21. LPS suppressed circVMA21 in BEAS-2B, while transfection of pcDNA 3.1-circVMA21 elevated circVMA21, as presented in Figure 2c. Adoption of MTT was to monitor cell viability. The suppression of LPS on the viability of BEAS-2B cells was attenuated via elevation of circVMA21, as proved in Figure 2d. The acceleration of LPS on BEAS-2B cell apoptosis was attenuated via augmentation of circVMA21 (Figure 2e). LPS treatment elevated inflammation in BEAS-2B cells, while augmentation of circVMA21 declined it (figure 2f). LPS treatment declined antioxidant enzymes SOD and GSH, but augmentation of circVMA21 recovered these two enzymes (Figure 2g). LPS boosted cleaved caspase-3 and phosphorylated NF-κB, and declined Nrf2, while augmentation of circVMA21 attenuated this action (Figure 2(h)). In brief, augmentation of circVMA21 was able to effectively ameliorate LPS-stimulated apoptosis and inflammation of BEAS-2B cell.

### 3.3 CircVMA21 competitively adsorbs miR-497-5p

**Figure 3.**
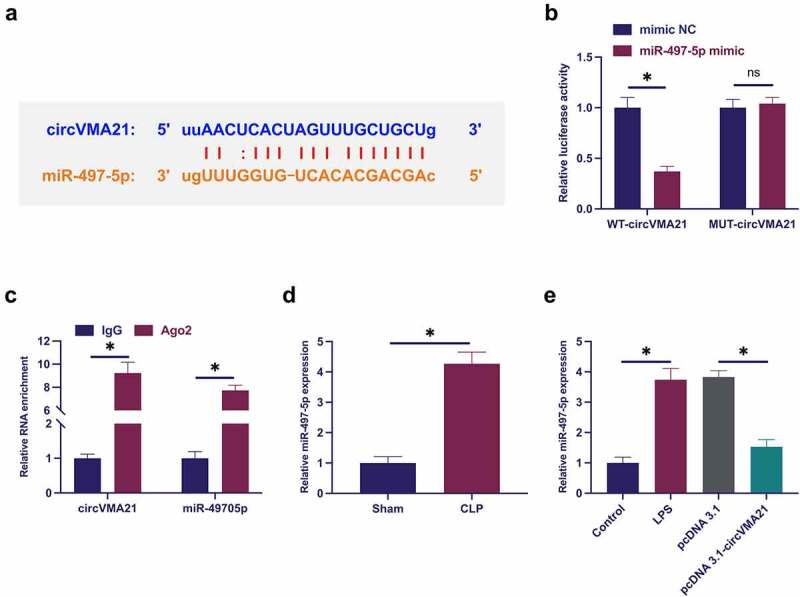
CircVMA21 competitively adsorbs miR-497-5p. (a): Prediction of binding site of circVMA21 and miR-497-5p via Bioinformatics website http://starbase.sysu.edu.cn/; (b): Verification of the targeting of circVMA21 with miR-497-5p via luciferase activity assay; (c): Validation of the binding of circVMA21 with miR-497-5p via RIP experiment; (d): Test of the influence of knockdown of circVMA21 on miR-497-5p in lung tissue of septic rats via RT-qPCR; (e): Examination of the impact of knockdown of circVMA21 on miR-497-5p in LPS-treated BEAS-2B cells via RT-qPCR; Representation of the data was as mean ± SD, N = 3; * *P*< 0.05.

The downstream targets of circVMA21 were figured out. Predicting potential binding sites of circVMA21with miR-497-5p was via http://starbase.sysu.edu.cn/ (Figure 3(a)). Co-transfection of WT-circVMA21 and miR-497-5p mimic reduced the luciferase activity, while co-transfection of MUT one exerted no influence on that (Figure 3(b)). CircVMA21 and miR-497-5p were abundant in the Ago2 vs. the IgG (Figure 3(c)). Subsequently, miR-497-5p in sepsis was examined. As presented in Figure 3(d,e), miR-497-5p was augmented in sepsis models, while elevation of circVMA21 turned around this phenomenon. In general, circVMA21 competitively adsorbed miR-497-5p.

### 3.4 Elevated miR-497-5p accelerates LPS-stimulated apoptosis and inflammation of BEAS-2B cell alveolar epithelial cells

**Figure 4.**
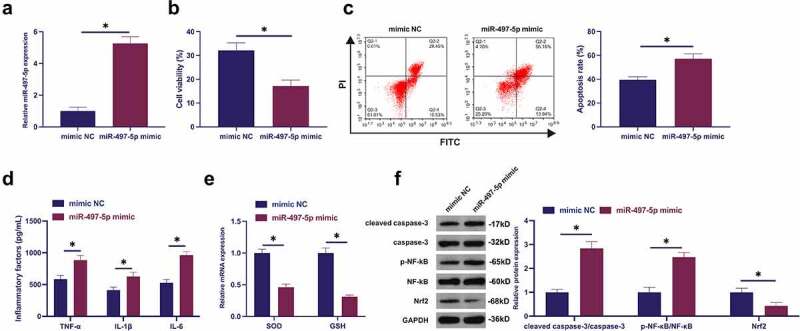
Augmented miR-497-5p facilitates LPS-stimulated apoptosis and inflammation of BEAS-2B cell alveolar epithelial cells. (a): Test of the transfection efficiency of miR-497-5p mimic via RT-qPCR; (b): Examination of the influence of elevated miR-497-5p on BEAS-2B cell viability via MTT; (c): Test of the impact of elevated miR-497-5p on the apoptosis rate of BEAS-2B cells via flow cytometry; (d): Examination of the impact of augmented miR-497-5p on inflammatory factors in BEAS-2B cells via ELISA; (e): Examination of the influence of augmented miR-497-5p on antioxidant enzymes in BEAS-2B cells via RT-qPCR; (f): Test of the influence of elevated miR-497-5p on cleaved caspase-3, Nrf2 and p-NF-κB in BEAS-2B cells via Western blot; Representation of the data was as mean ± SD, N = 3; * *P* < 0.05.

The biological function of miR-497-5p in septic LI was explored. Transfection of miR-497-5p mimic resulted in the augment of miR-497-5p (Figure 4(a)). Elevated miR-497-5p further reduced cell viability and increased apoptosis rate (Figure 4(b,c)). Elevated miR-497-5p augmented inflammation (Figure 4(d)). SOD with GSH was declined after elevation of miR-497-5p (Figure 4e). Augmented miR-497-5p elevated cleaved caspase-3 and phosphorylated NF-κB, and Nrf2 was declined (figure 4f). In short, elevated miR-497-5p boosted LPS-stimulated apoptosis and inflammation of BEAS-2B cell alveolar epithelial cells.

### 3.5 MiR-497-5p targets CD2AP

**Figure 5.**
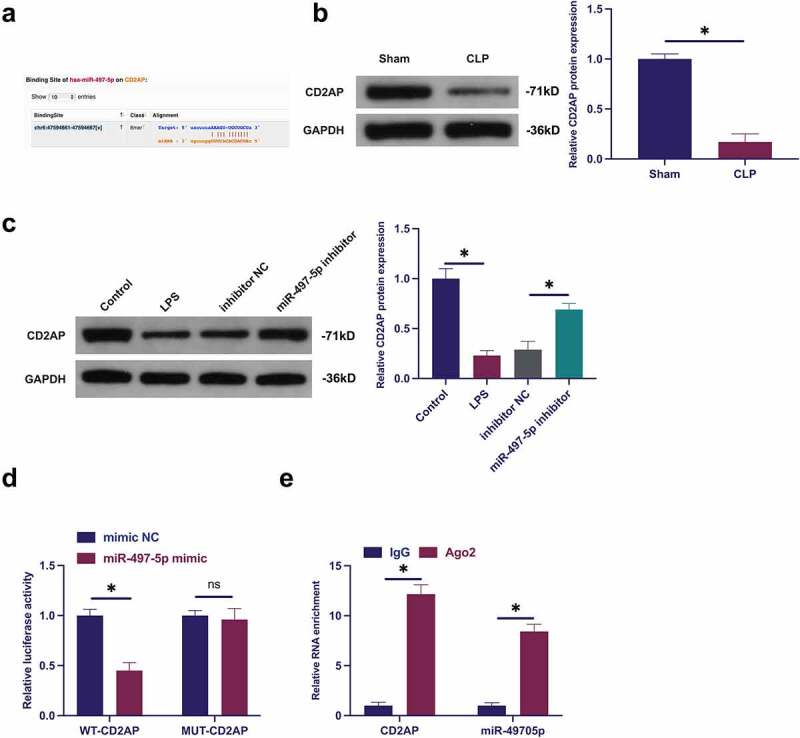
MiR-497-5p targets CD2AP. (a): Forecast of binding sites of CD2AP and miR-497-5p via Bioinformatics website http://starbase.sysu.edu.cn/; (b): Test of CD2AP in septic rats via Western blot; (c): Examination of the impact of knockdown of miR-497-5p on CD2AP in BEAS-2B cells via Western blot; (d): Validation of the targeting of CD2AP with miR-497-5p via deal luciferase report experiment; (e): Verification of the binding of circVMA21 with CD2AP via RIP experiment; Representation of the data was as mean ± SD, N = 3; * *P* < 0.05.

The target genes of miR-497-5p were explored. CD2AP with miR-497-5p was provided with potential-binding sites through screening (Figure 5(a)). Additionally, CD2AP was declined in lung tissue and LPS-treated BEAS-2B cell of septic rats, while repressive miR-497-5p distinctly recovered CD2AP (Figure 5(b,c)). Consequently, miR-497-5p was supposed to target CD2AP. Meanwhile, several experiments were carried out to prove this conjecture (Figure 5(d,e)). The results elucidated that co-transfection of WT-CD2AP and miR-497-5p mimic silenced luciferase activity, while co-transfection of MUT one acted no influence on luciferase activity. Besides, CD2AP with miR-497-5p was abundant in Ago2 magnetic beads. In short, miR-497-5P targeted CD2AP.

### 3.6 CircVMA21 ameliorates LPS-stimulated apoptosis and inflammation of BEAS-2B cell via modulating the miR-497-5p/CD2AP axis

**Figure 6.**
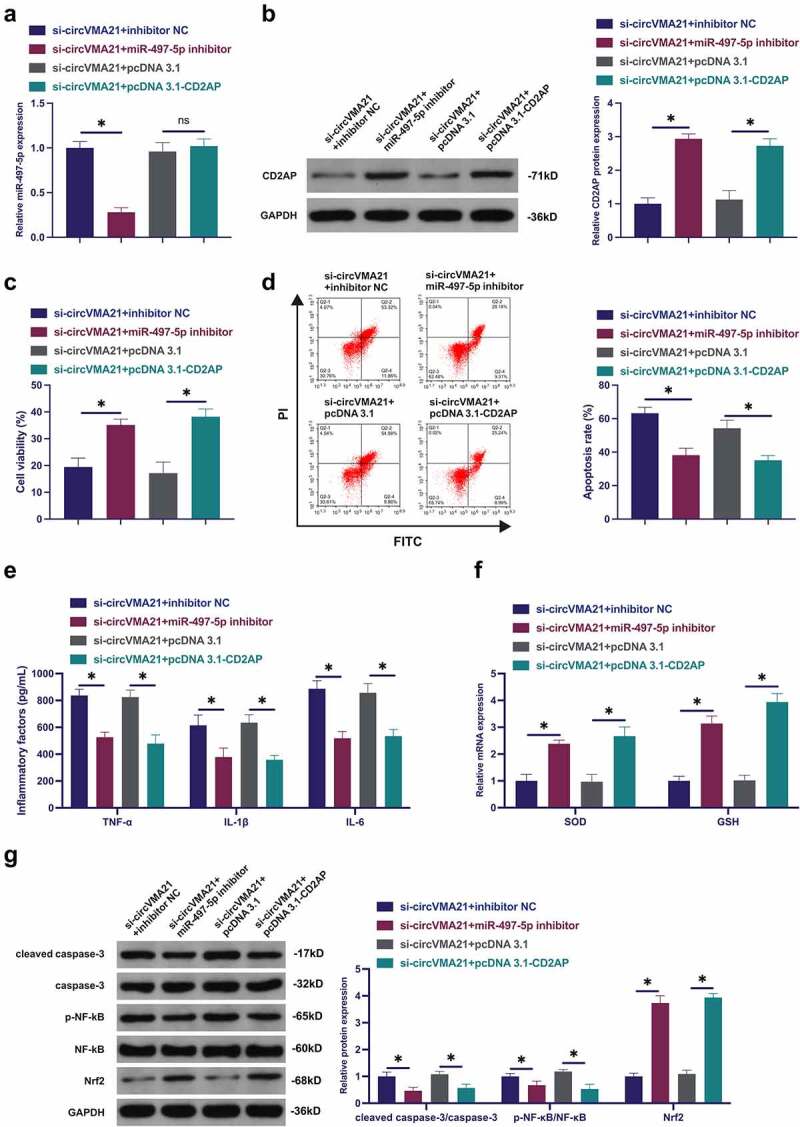
CircVMA21 ameliorates LPS-stimulated apoptosis and inflammation of BEAS-2B cell via modulating the miR-497-5p/CD2AP axis. (a): CircVMA21 was knocked down, while silenced miR-497-5p or elevated CD2AP exerted an influence on miR-497-5p in BEAS-2B cells; (b): CircVMA21 was knocked down, while silenced miR-497-5p or elevated CD2AP exerted an influence on CD2AP in BEAS-2B cells; Assessment of the influence of knocking down circVMA21 and silenced miR-497-5p or elevated CD2AP on BEAS-2B cell viability via Function rescue assay (c), Apoptosis rate (d), Inflammatory factors (e), The influence of antioxidant enzymes (f) and cleaved caspase-3, p-NF-κB, and Nrf2 (g); Representation of the data was as mean ± SD, N = 3; * *P*< 0.05.

Subsequently, the mechanism of circVMA21/miR-497-5p/CD2AP axis impacting LPS-stimulated alveolar epithelial cell BEAS-2B cell injury was explored. When circVMA21 was knocked down, miR-497-5p was silenced or CD2AP was elevated. Knocking down circVMA21 accelerated miR-497-5p, while knocking down miR-497-5p was turned around in the meantime (Figure 6a)). Silenced circVMA21 suppressed CD2AP, while repressive miR-497-5p or elevation of CD2AP turned around this phenomenon (Figure 6(b)). Silenced circVMA21 restrained the cell viability, and cell apoptosis was boosted, while knockdown of miR-497-5p or augmentation of CD2AP attenuated this action (Figure 6(c,d)). Knocking down circVMA21 accelerated inflammation, and GSH and SOD were declined, while silenced miR-497-5p or elevated CD2AP turned around this phenomenon (Figure 6(e,f). Knocking down circVMA21 elevated cleaved caspase-3 and phosphorylated NF-κB, and Nrf2 was declined, while silenced miR-497-5p or augmented CD2AP prevented the variation of these proteins (Figure 6g). To sum up, circVMA21 was available to ameliorate LPS-stimulated apoptosis and inflammation of BEAS-2B cell via modulating the miR-497-5p/CD2AP axis.

## 4 Discussion

Sepsis, a systemic inflammatory response caused via microbial infection [20], is available to trigger multiple organ dysfunction, primarily covering lungs and kidneys [21]. The treatment methods for sepsis were provided with certain limitations till now, and the morbidity and mortality were unable to be tellingly declined. Consequently, early diagnosis and progression of brand-new cure methods were vital for the treatment of sepsis. CircRNA, a specific endogenous RNA, is implicated in multiple illnesses’ pathogenesis as a gene regulatory factor [22], moreover, circRNA is available to be adopted as the potential biomarkers of cancer [23], skin diseases [24] and nervous system disease [25]. Studies have been elucidated that LI caused via sepsis is linked with inflammation, covering lung cell apoptosis and the generation of inflammatory cytokines [26], and these processes were modulated via circRNA.

In this study, the influence of circVMA21 on CLP-stimulated LI in a mouse model of sepsis and LPS-stimulated apoptosis and inflammation of BEAS-2B cell was explored. Cecal ligation and puncture (CLP), a prevalently adopted in *vivo* sepsis model, is available to represent the human condition [27]. The CLP model adopts immunoactive rat for surgical suture and ligation of the cecum, resulting in ischemia and necrosis, and then the cecum is punctured, which the cecal contents overflow to the peritoneum, leading to local infection and peritonitis, and ultimately organ failure was performed [28]. LI is the extremely prevalent organ dysfunctions in sepsis. In this study, elevated circVMA21 recovered the survival rate of CLP rats, declined lung water content, lung cell apoptosis and inflammation, and mitigated LI, while knocking down circVMA21 was in the opposite. These results clarified that circVMA21 exerted a critical role in sepsis-stimulated lung tissue damage.

Numerous evidences elucidated that oxidative stress boosted sepsis progression [29], which is available to trigger impaired vascular permeability, declined heart function, and mitochondrial dysfunction leading to impaired breathing, and ultimately generate multiple-organ dysfunction and death [30]. Mitochondrial dysfunction and reactive oxygen species (ROS) generated via mitochondria exerts a role in the pathogenesis of sepsis [31]. Foregoing studies have been maintained that CLP triggers the accumulation of ROS, and suppresses the activity of superoxide dismutase (SOD), which manifests that oxidative stress is augmented in septic rat [32]. Consequently, the influence of circVMA21 on oxidative stress in sepsis was explored. LPS treatment declined antioxidant enzymes SOD and GSH, but elevated circVMA21 turned around the performance of LPS. CircVMA21 aggravated oxidative stress in sepsis advancement. The transcription factor Nrf2 is the vital regulator of antioxidant and detoxification cellular responses [33]. Studies have been elucidated that activation of Nrf2 is available to suppress the elevation of NF-κB on pro-inflammatory cytokines [34]. As reported, excessive inflammation is the critical feature of sepsis and leads to premature death of patients. In this study, elevated circVMA21 accelerated Nrf2, and suppressed the phosphorylation of NF-κB and inflammation, while silenced circVMA21 was opposite. The above results clarified that elevated circVMA21 was available to ameliorate oxidative stress and inflammation in septic rat. Caspase-3 is the vital executor of the apoptosis, and is augmented in the serum of sepsis patients [35]. In this study, elevated circVMA21 restrained cleaved caspase-3. That was, augmented circVMA21 alleviates sepsis-stimulated lung cell apoptosis.

Additionally, the molecular mechanism of the action of circVMA21 in sepsis-stimulated LI is explored, which manifests that miR-497-5p is augmented in sepsis models. Elevated miR-497-5p accelerates LPS-stimulated apoptosis and inflammation of BEAS-2B cell, which is consistent with the findings of foregoing researches, while augmented circVMA21 represses miR-497-5p and turns around elevated miR-497-5p’s facilitation on sepsis-stimulated LI. Subsequently, miR-497-5p’s target gene was explored, and the latent-binding sites with CD2AP were discovered. CD2-associated protein is the crucial proteins modulating cytoskeleton assembly and cell adhesion [36]. A previous study has showed CD2AP binds to intercellular adhesion molecule-1 to regulate mechanical signal transduction, leukocyte adhesion, and exerts an important role in kidney inflammation [37]. Studies have been elucidated that CD2AP is silenced in acute renal failure caused via LPS-stimulated sepsis [38]. In this study, CD2AP is declined in lung tissue and cells in septic rats, which is consistent with the findings of foregoing researches, while silenced miR-497-5p tellingly recovered CD2AP. It is noteworthy that multiple studies have manifested changes in CD2AP and TLR4/NF-κB inflammatory signaling pathway are co-existed during inflammatory injury [39,40]. Therefore, we speculated the TLR4/NF-κB inflammatory signaling pathway may be a downstream regulatory pathway of CD2AP, which needed to be explored in subsequent studies. The experiments manifested that CD2AP was miR-497-5p’s target. Additionally, the acceleration of silenced circVMA21 on septic LI was alleviated via knockdown of miR-497-5p or augmentation of CD2AP. In brief, circVMA21 ameliorated LPS-stimulated apoptosis and inflammation of BEAS-2B cell via controlling the miR-497-5p/CD2AP axis.

## 5 Conclusions

The beneficial role of circVMA21 in *vivo* and in *vitro* models of sepsis is explained in this study, but there are still significant barriers to clinical transformation. Since late sepsis has a high mortality rate, determinating whether circVMA21 can be used as a biomarker for early sepsis diagnosis will be beneficial for monitoring and warning the development of sepsis. In addition, the clinical manifestations of different sepsis are heterogeneous, and it is distinct from those of rat or cellular sepsis models. The dose and efficacy of lentiviral plasmids of delivery circRNA might be limited by complications in *vivo*. CircRNA network is a complex and large mechanism, so a single circRNA regulation might have limited action in human body, and it is a necessity to conduct a more comprehensive and in-depth study on the regulation mechanism of circRNA later. To sum up, elevated circVMA21 suppresses oxidative stress, apoptosis and inflammation via mediating the miR-497-5p/CD2AP axis to mitigate LI in septic rat. The data offers a theoretical basis for CircVMA21 as a latent therapeutic target for the treatment of sepsis-stimulated LI later.
